# Recommendations from the IRDiRC Working Group on methodologies to assess the impact of diagnoses and therapies on rare disease patients

**DOI:** 10.1186/s13023-022-02337-2

**Published:** 2022-05-07

**Authors:** Galliano Zanello, Chun-Hung Chan, David A. Pearce, IRDiRC Working Group

**Affiliations:** 1 IRDiRC Scientific Secretariat, INSERM, Paris, France; 2 Sanford Research, Sioux Falls, SD 57104 USA; 3 Sanford Health, Sioux Falls, SD 57117 USA; 4 Sanford School of Medicine, University of South Dakota, Sioux Falls, SD 57105 USA

**Keywords:** Rare diseases, Diagnosis, Therapies, Socio-economic impact, Patients, Families, Care givers, Health care systems, Payers

## Abstract

Rare disease patients face many challenges including diagnostic delay, misdiagnosis and lack of therapies. However, early access to diagnosis and therapies can modify the management and the progression of diseases, which in return positively impacts patients, families and health care systems. The International Rare Diseases Research Consortium set up the multi-stakeholder Working Group on developing methodologies to assess the impact of diagnoses and therapies on rare disease patients. Using the patients’ journey on the diagnostic paradigm, the Working Group characterized a set of metrics, tools and needs required for appropriate data collection and establishment of a framework of methodologies to analyze the socio-economic burden of rare diseases on patients, families and health care systems. These recommendations are intended to facilitate the development of methodologies and to better assess the societal impact of rare diseases.

## Background

Rare diseases, defined as affecting < 200,000 in the US or fewer than 1 in 2000 people in the European Union, are uncommon when considered individually but with as many as 7000 rare diseases[1], collectively they are estimated to affect more than 300 million individuals worldwide and up to 30 million in the European Union [2]. This equates to 3.5–5.9% of the world’s population, or between 1 in 17 to 1 in 29 people, and therefore it is evident that taken together, rare diseases are actually quite prevalent. Despite this, diagnosis and treatment of rare diseases often lags behind that of common diseases due to the vast number of different disorders and the small number of patients with these individual disorders. Many times, patients with rare diseases will undergo prolonged diagnostic journeys, termed the diagnostic odyssey, in order to arrive at an accurate diagnosis [3–5]. Even after a diagnosis is finally made, patients with rare diseases can often find that there are few options, if any, for treatment and when treatments exist, they can be very expensive. Approvals for rare disease drugs has accelerated significantly in recent years, in part because of the passage of the US Orphan Drug Act in 1983. As of January 1, 2020, a total of 564 orphan drugs have been approved by the US Food and Drug Administration (FDA) to treat 838 rare diseases [6]. In the European Union, as of March 2021, 127 medicinal products have an orphan designation with market authorization while 260 medicinal products intended for rare diseases have a market authorization without European orphan designation [7]. However, this is only a small fraction of the approximately 7,000 known rare diseases leaving many without viable treatments.

In February 2017, the International Rare Diseases Research Consortium (IRDiRC) defined it’s vision and goals to achieve by the year 2027: *Enable all people living with a rare disease to receive an accurate diagnosis, care, and available therapy within one year of coming to medical attention.* Three new goals were set (https://irdirc.org/about-us/vision-goals/):

- *Goal 1*: All patients coming to medical attention with a suspected rare disease will be diagnosed within one year if their disorder is known in the medical literature; all currently undiagnosable individuals will enter a globally coordinated diagnostic and research pipeline
- *Goal 2*: 1000 new therapies for rare diseases will be approved, the majority of which will focus on diseases without approved options
- *Goal 3*: Methodologies will be developed to assess the impact of diagnoses and therapies on rare disease patients

As such, Working Group on Goal 3 (WG3) was established to evaluate the specific needs, metrics and tools for addressing the third goal of IRDiRC: *Develop methodologies to assess the impact of diagnoses and therapies on rare disease patients*. IRDiRC members nominated experts across many disciplines that could contribute their knowledge to efficiently execute this goal. The members of the WG3 are shown in Table 1. The WG3 met in Paris, France in February 2020 and the results of those discussions are reported here.

**Table 1:** Members of the IRDiRC Working Group on Goal 3 (WG3)

Name	Affiliation
Patrizio Armeni	SDA Bocconi School of Management, Milan, Italy
Dimitrios Athanasiou	MDA Hellas, Athens, Greece
Alicia Bauskis	Department of Health, Western Australia, Perth, Australia
John Belmont	Illumina, San Diego, CA, USA
Anna Bucsics	Mechanism of Coordinated Access to orphan medicinal products (MoCA), Vienna, Austria
Peter Fish	Mendelian, London, UK
Josie Godfrey	JG Consulting, London, UK
Daniel Ollendorf	Tufts Medical Center, Boston, MA, USA
Manuel Posada	Institute of Rare Diseases Research, Institute of Health Carlos III, Madrid, Spain
Michael Schlander	University of Heidelberg, Heidelberg, Germany
Vicky Seyfert-Margolis	My Own Med, Inc., Bethesda, MD, USA
David A. Pearce	Sanford Health, Sioux Falls, SD, USA
Galliano Zanello	IRDiRC Scientific Secretariat, Inserm, Paris, France

## Methods

A call for nominations was opened to the IRDiRC membership to identify qualified individuals to form the IRDiRC WG3. Members of the WG3 were selected based on expertise, with thought given to ensure that the group was balanced and had complementing expertise. Members of the WG3 (Table 1) comprised an international panel with expertise in health economics, patient advocacy, genetics, health technology assessment (HTA), population health, epidemiology, reimbursement, diagnoses, drug evaluation, health care interventions, value measurements, and health policies. While we had representation from many developed nations worldwide, one key limitation was the lack of representation from developing nations which could limit generalizability of the framework that was developed, particularly in developing nations.

Prior to the in-person discussion, 6 conference calls were held to set the agenda and key discussion points. The WG3 convened in Paris, France over two days in February of 2020 to discuss and develop the framework for developing methodologies to assess the impact of diagnoses and therapies on RD patients. The WG3 engaged in discussion to analyze how access to diagnostics and therapies impact the economy of health systems, as well as the socio-economic burden on rare disease patients and their families. At discussion were the requirements for developing methodologies to assess the impact of diagnoses and therapies on RD patients, and a proposed framework for implementing. The results of these discussions were compiled and presented to the IRDiRC Consortium Assembly in March 2021 where comments from all members of IRDiRC in attendance were solicited.

## Results

### Assessing the impact of diagnosis and therapies on rare disease patients

The diagnostic journey is often cited for virtually all rare diseases. Patients and families are desperate to know the underlying cause or sometimes even just the name of the disease or condition they are afflicted with. In a recent study conducted in Canada and the United Kingdom, parents of patients with undiagnosed or rare disease were surveyed to determine the information that they value the most. As expected, parents valued receiving a causal diagnosis as well as management strategies to improve patient health, but also valued expanding research to broaden the evidence base upon which diagnoses could be made quickly and accurately [8]. It is therefore evident that improvements in diagnosis of rare diseases is an important factor in meeting the needs of rare disease patients and their families.

**Fig. 1:**
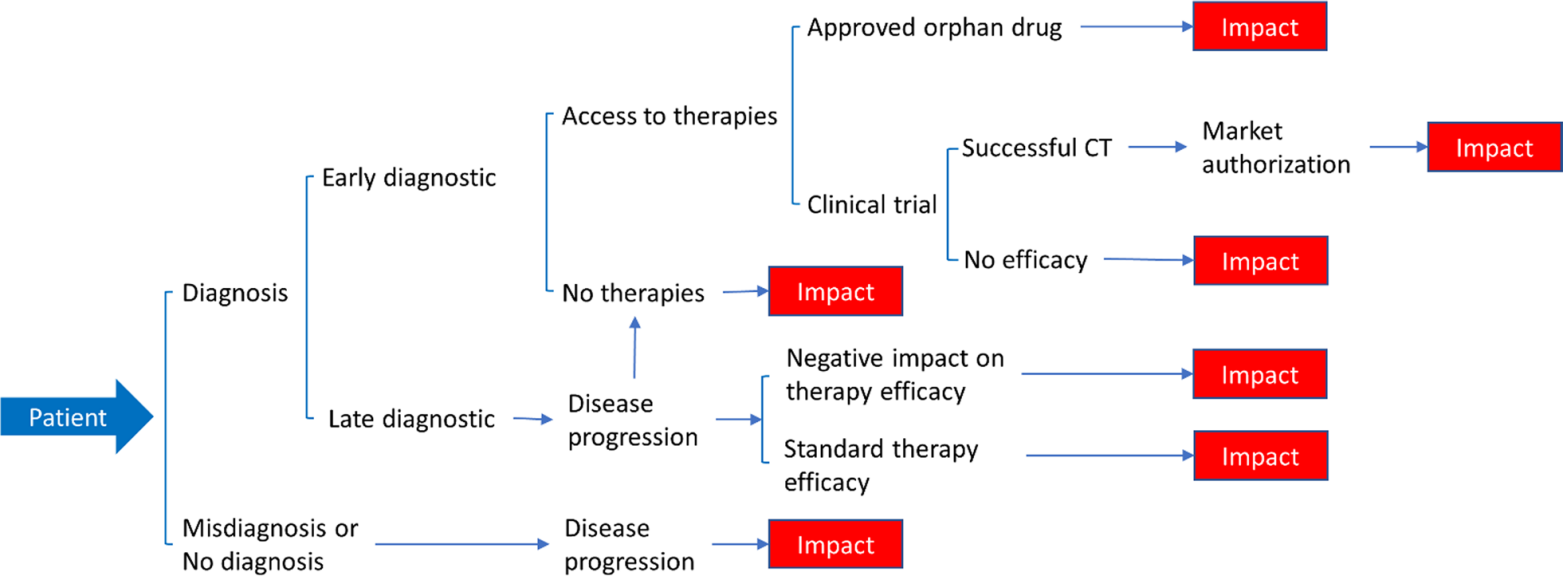
A patient’s journey on the diagnostic paradigm and areas of impact. A simplified schematic of the patient’s journey showing the various scenarios that may arise and highlighting the important areas of impact for patients. Please note that while this schematic shows a linear journey for RD patients, there are instances where a patient may return to an earlier point in their journey, perhaps due to changes in diagnostic technology, understanding of the disease, or lack of positive outcomes with approved therapies

To understand the impact of diagnoses and therapies on RD patients, we first need to view the journey from the patient perspective. Insight into patient experiences was provided by not only the patient advocate representative on WG3, but also by other members of WG3 who have had extensive experience working with RD patients and have an understanding of the struggles they face. In addition, challenges faced by RD patients is well documented and widely understood by those in the RD community [3–5, 9–12] In Fig. 1, we have mapped the diagnostic paradigm from the patient’s perspective and the various directions it may take. While this is a simplified schematic of possible pathways, we acknowledge that, in reality, the diagnostic/therapeutic journey for the RD patient can be much more complex and will include social impact associated with diagnosis and/or treatment. In many cases, the patient’s path to receiving a definitive diagnosis can be long and is often referred to as a diagnostic odyssey that can take many years and require travel to specialists located far from home [4, 5, 8–11]. As shown in Fig. 1, the patient’s journey often ends in a situation that negatively impacts their health and wellbeing, as well as that of their families. This often occurs when there is misdiagnosis/no diagnosis, a diagnosis but no approved therapies, disease progression despite therapy, and when clinical trials fail to show efficacy. Despite these negative impacts, it is also important to highlight paths that lead to positive impacts such as when there are approved therapies available after diagnosis with efficacy and successful clinical trials leading to market approval. It should be noted that while access to approved therapies or successful clinical trials may be positive impacts on RD patients, it is also possible that these therapies may not successfully treat individual patients causing them to return to an earlier point in their journey where an effective therapy may not be available. By charting the outcomes based upon the patient’s journey, it is possible to use this diagram as a template for evaluating the impact of improved diagnostics and the ultimate development of therapies.

While improved health and wellbeing is the ultimate goal for rare disease patients and their families, it is important to also consider the socio-economic impact of living with a rare disorder [13–16]. Individuals living with a rare disease, and those who care for them, can often suffer from social stigmas, as well as being unable to participate fully in everyday activities. This is often coupled with increased economic burden brought about by having a rare disease, even when effective treatments are available [17].

Access to diagnosis and therapies can influence the management and the progression of diseases which can impact not only patients and their families, but also health care systems (HCS) that provide their care. Patients with rare disease can pose a challenge to most health care systems that are not equipped to provide the specialized diagnostics and care that are needed, particularly in countries that lack rare disease programs or robust referral systems to specialists. However, even in countries with access to rare disease programs or advanced diagnostics, RD patients can still face challenges that may be related to costs, lack of awareness, or insurance. This can lead to increased healthcare expenditures impacting not only patients, but also HCS and providers, thus reducing the efficiency of care. In addition, the increased burden on health providers, long diagnostic odysseys, the need for specialized care, and the high price of approved treatments can result in disproportionately high health care costs [18, 19]. These costs are often borne by the patient/families, health insurers or in countries where social medicine is provided, the government. Thus, it is important to also consider the burden of rare diseases on not only patients/families and HCS, but also on health insurers and/or government programs. In a report released by the EveryLife Foundation for Rare Diseases, the economic cost of 379 rare diseases in the USA in 2019 was estimated to nearly 1 trillion USD when including indirect costs such as loss of productivity and non-medical and other uncovered healthcare costs [15].

**Fig. 2:**
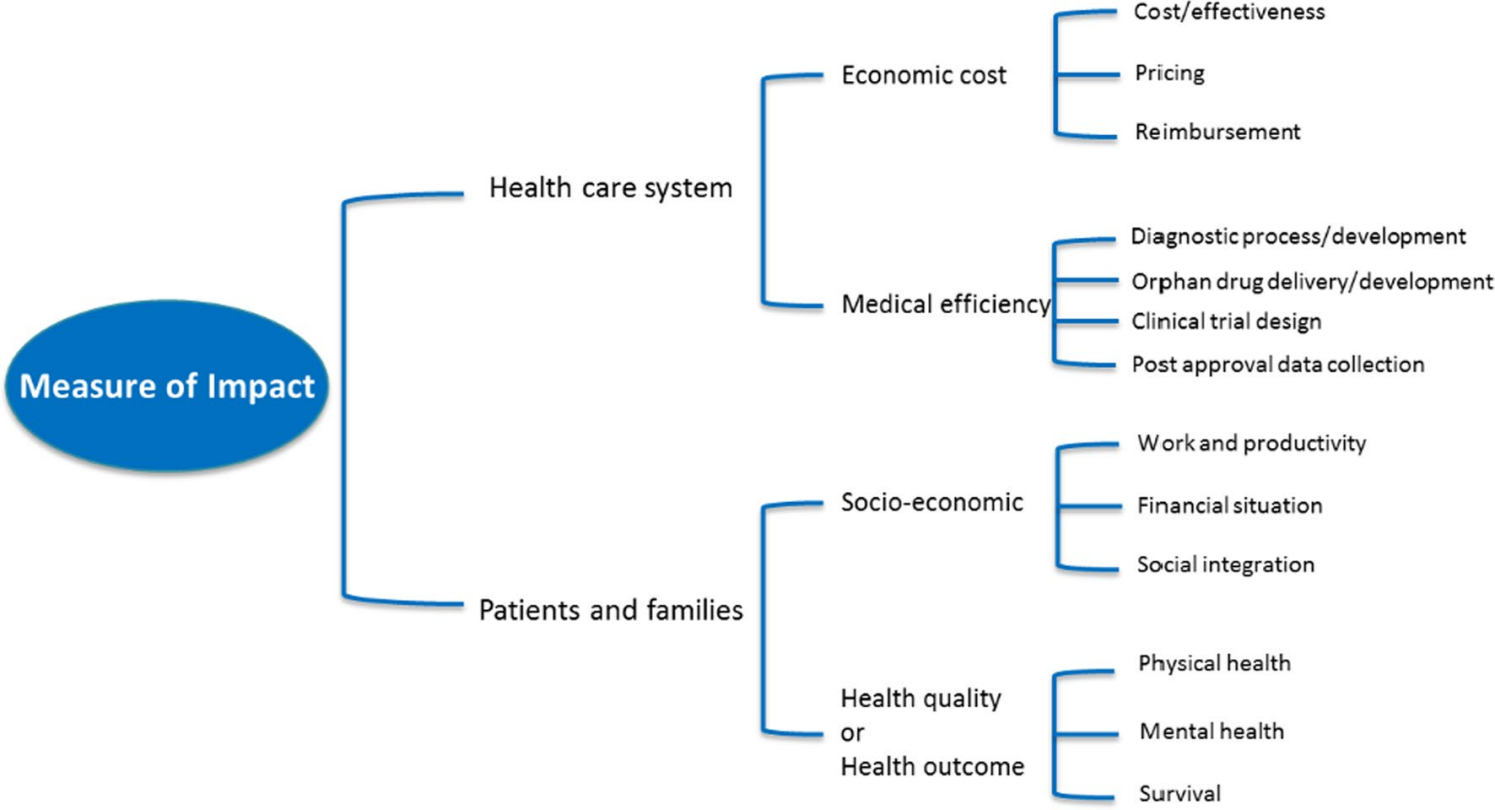
Measure of impact of diagnostics and therapies for patients, families and health care systems. This graphic shows how access to diagnoses and therapies impacts the quality of life of patients and families, and also the economics and efficiency of health care systems

The WG3 identified that the development of methodologies is dependent on the preliminary selection of metrics highlighting how access to diagnostic and therapies impact the health quality of rare disease patients, the socio-economic burden on patients and families, and the economy and efficiency of HCS and insurance companies. For patients and families, the most important factors to consider are quality of life (QoL) or health outcomes and the socio-economic burden of RD. When considering QoL/health outcomes, one must consider not only the physical health (and survival) but also the mental health of patients and/or their families, while socio-economic factors such as work/productivity (particularly for caregivers), finances, and social integration are also important. In contrast, HCS must consider both the economic cost and the medical efficiency of providing care to RD patients. To provide the best care for patients, HCS must consider the diagnostic process, delivery of treatments, clinical trials where approved treatments are not available, and collection of patient outcome data. These must be balanced with the economic cost associated with RD patients, such as reimbursement for tests or drugs that are not covered, the high cost of therapeutic drugs for RD, and the overall cost effectiveness of treating each patient (Fig. 2 and Table 2). The categories presented in Fig. 2 and Table 2 should form the basis for further discussions as new methodologies are developed. One key element that was not included in these categories is the concept of budget impacts on HCS. While budget impacts on both HCS and insurers was discussed, it was beyond the scope of the WG3 given the complexities of budget analysis in healthcare. It is hoped that a future IRDiRC task force will be convened to tackle the issues surrounding budget impacts and the overall economics of providing care to RD patients. Additionally, we have not included other potential outcomes related to HCS and insurers, such as research advances and innovation, primarily as there is still a significant need to engage these stakeholders in activities such as enhancing use of electronic medical records to identify RD patients, support registries, or natural history studies, expanding the use of advanced diagnostics, and developing better clinical decision support tools. We hope that by providing an initial starting point for HCS and insurers to understand the issues surrounding RD patient care, we can engage them fully in all aspects of RDs including research, patient/family support, and societal impacts.

**Table 2:** Combined metrics for both patients and providers for rare diseases

Patients and their families	Health Systems
Health quality and outcome Physical health and disease progression Mental health Survival Socio-economic burden Work and productivity Financial status Social integration Time availability	Economic costs and efficiency of delivery Integrate the quality of the health care service into economic analysis (cost effectiveness, pricing, reimbursement) Compare the rising cost of diagnosis and the benefit of treating patients early on or at a later stage, and how it affects treatment efficacy and disease progression Develop cost effectiveness criteria based on social preferences (quality of life, social value) for the conventional evaluation of medical components Evaluate the impact of access to diagnosis on the cost of clinical components such as appropriate decision making by the physicians, appropriate therapy delivery, appropriate study design and development of end points in clinical trials

**Fig. 3:**
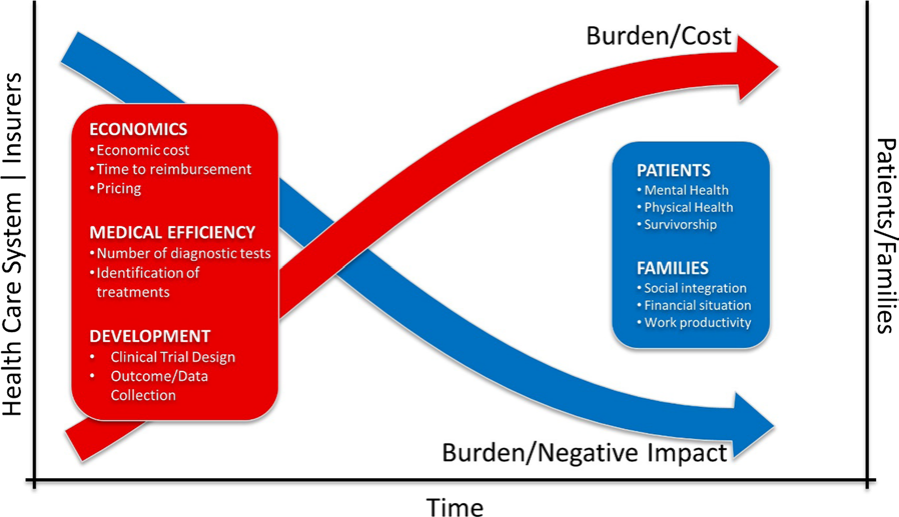
Evolution of the disease burden and the economic cost over time for all stakeholders. This graphic represents how decrease in negative health and socio-economic outcomes on patients and families are associated with an increase of the burden and economic costs on health care systems and payers

As we consider the impact of RD for both patients/families and HCS, it should be noted that the factors mentioned above are closely linked, which we hypothesize to be an inverse relationship between the burden/negative impact on patients/families and the burden/cost on HCS. This is illustrated in Fig. 3 which shows that over time, as the burden and cost increases for the HCS as they provide continued care, the burden and degree to which the RD negatively impacts a patient/family decreases as they receive treatment. We acknowledge that this is a very simplistic view of the burden of rare diseases and that there are many factors that will influence both the on-going costs to the HCS/Insurer and the burden/negative impact on the patient and their families. This is not intended to be used as evidence on which to base decisions on whether investments in rare diseases will provide a good return, but rather, is intended to illustrate that there is a casual relationship between these very important factors. Indeed, as we consider the impact on HCS and insurers, it is clear that budgetary considerations often drive business decisions given market pressures, increasing cost of healthcare, and lack of funding. However, it is essential for HCS and insurers to also consider that investments in RDs could lead to lower expenditures, as well as providing direct benefits to the patients, and an indirect societal benefit from expanding the RD knowledgebase.

Although the concepts above appear to be at odds, it should be noted that this is presented from the viewpoint of the various stakeholders involved in the WG3. By identifying the key metrics with which to develop effective tools for measuring the impact of rare diseases, it may be possible to re-align these concepts such that they are synergistic and mutually beneficial. For instance, improvements in care and QoL for the patient does not always equate to increased costs for the HCS/insurer. In fact, in some instances, costs may actually decrease as a result of better disease management and a reduction in healthcare utilization. Therefore, it is important to ensure that HCS and insurers are positioned to work alongside researchers and advocacy groups to collectively understand how the burden of RDs can be reduced, whilst ensuring benefits for all stakeholders.

### Meeting the needs of rare disease patients

**Fig. 4:**
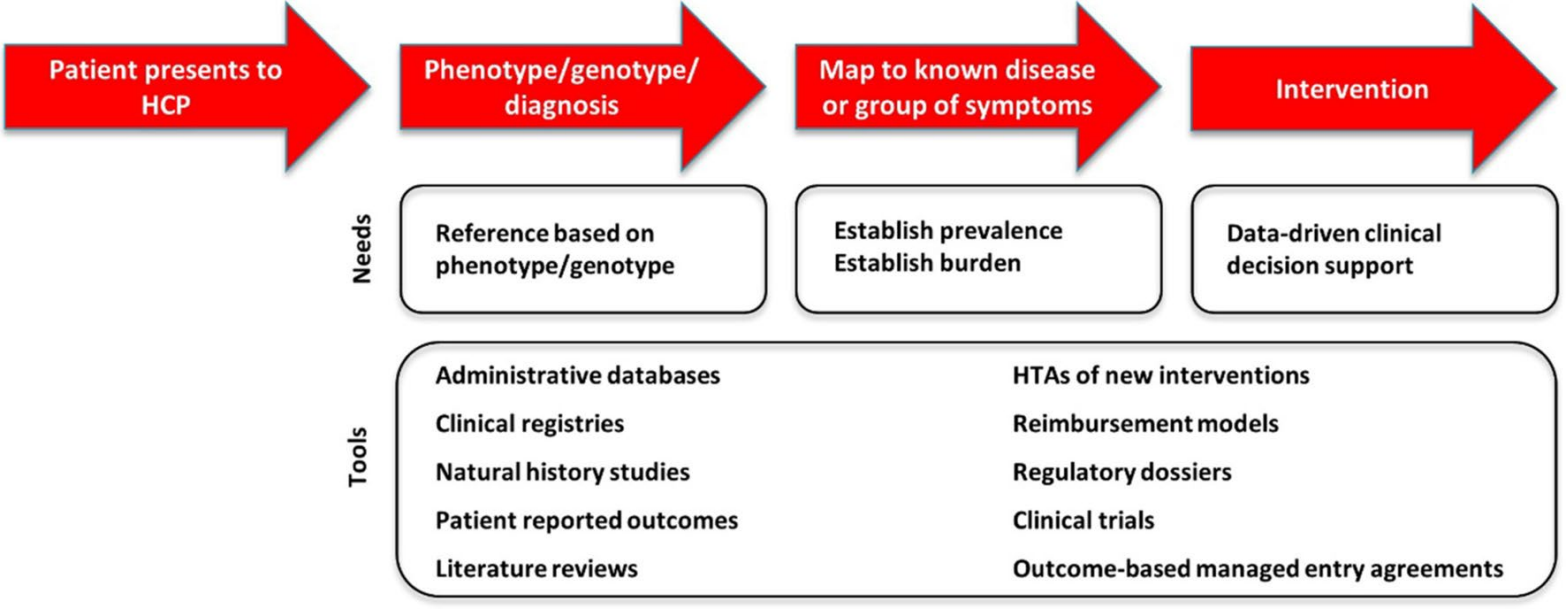
Proposed data that is needed to define the patient journey from the perspective of healthcare providers and systems. This graphic summarizes the patient journey from diagnostic to clinical intervention, the needs at each step of this journey and the data sources and/or tools that can be used or developed to measure the impact of diagnosis and therapies on patients and health care systems

It is evident that the needs of rare disease patients are currently unmet and much work remains to address the long diagnostic journeys encountered by patients, the lack of treatments for many diseases, and the high socio-economic burden on patients, their families, HCS, health insurance companies, and governments. To address those unmet needs, and to achieve the third goal of IRDiRC: *Develop methodologies to assess the impact of diagnoses and therapies on rare disease patients*, we have defined the needs along each step of a patient journey, and the tools from which those needs could be met. Figure 4 summarizes the patient journey from diagnostic to clinical intervention, the needs at each step of this journey and the data sources and/or tools that can be used or developed to measure the impact of diagnosis and therapies on rare disease patients and the HCS. Ideally, upon first presentation to a health care provider (HCP), the patient will be examined and phenotyped. In practice, the initial examination is likely to be superficial and a detailed examination with appropriate phenotyping will most likely not occur. Along the way, the patient may visit multiple providers before a more detailed examination and phenotyping occurs. Depending on the presentation of symptoms, the HCP may make a diagnosis based on phenotype alone or order genetic testing to either confirm diagnosis or to make a diagnosis. The greatest need at this early stage in a patient’s journey are robust data on phenotypes and genotypes associated with disease. The next stage of a patient’s journey once a diagnosis is made, if one is made, is to map to known diseases or groups of symptoms in order to understand how to provide appropriate care. The need at this point is to establish both the prevalence of the disease, as well as the burden of disease. Once a patient receives a diagnosis, a treatment plan can be developed and intervention can begin. The most important need at this stage is data-driven clinical decision support to guide the HCP in making treatment decisions based on the patient’s presentation. To address these needs, a number of different data sources can be utilized including databases, such as administrative databases, electronic medical records, clinical registries, natural history studies, patient reported outcomes, literature reviews, health technology assessments of new interventions, regulatory dossiers, clinical trials, reimbursement models, and outcomes-based managed entry agreements. By accessing these data sources, it should be possible to build the necessary tools to support the HCP in patient managements, and by extension the same data can be used to measure the impact of diagnosis and therapies on both the patient and the HCS.

**Fig. 5:**
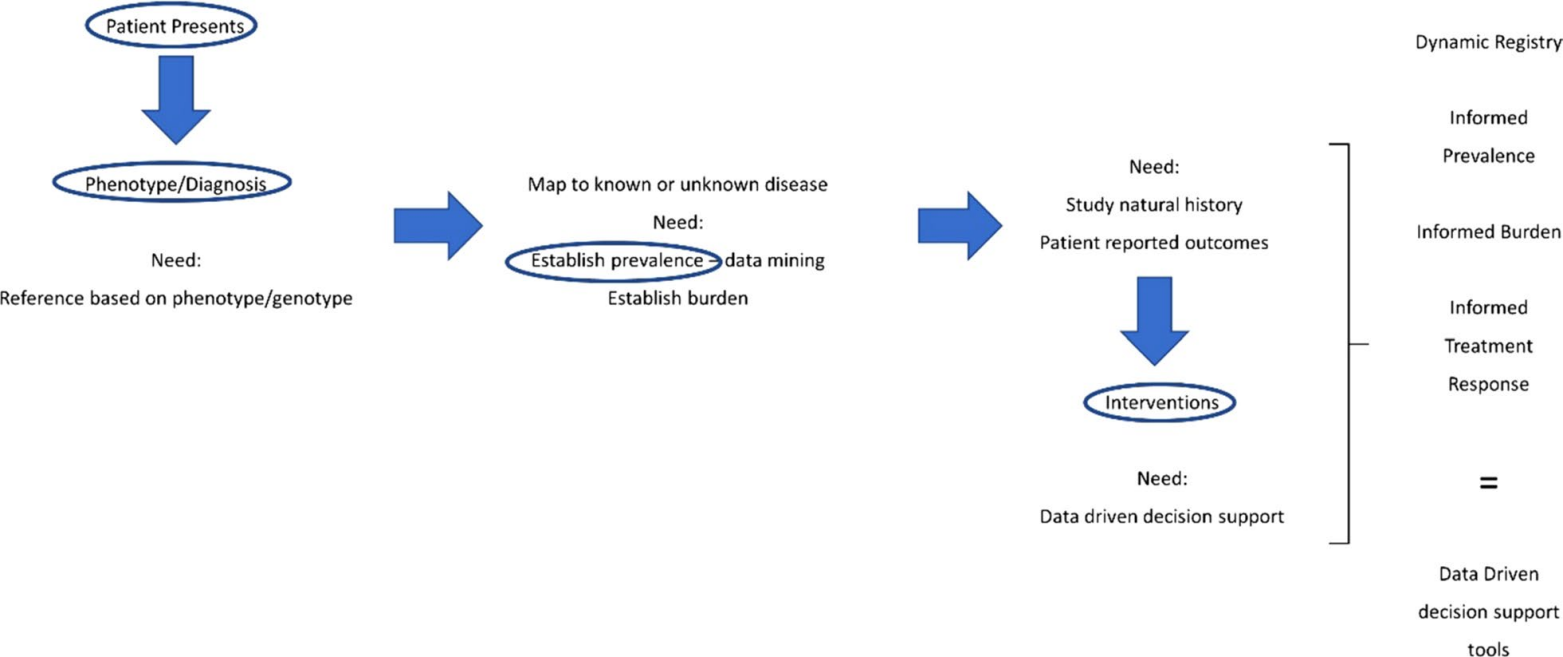
The patient journey as seen from the perspective of healthcare providers and systems. This graphic represents the patient needs for efficient diagnosis and clinical intervention, and the area for which data or tools are needed.

Developing methodologies to assess the impact of diagnosis and therapies on rare disease patients requires identifying the needs of the patient themselves. The WG3 developed a template representing the patient journey with respect to diagnosis and therapies. In Fig. 4, the group characterized the needs for efficient diagnosis and intervention and the area for which data or tools are needed. Figure 5, represents the same needs identified in Fig. 4 in a more succinct manner. The highlighted areas represent the areas where data is absolutely necessary in order to develop data driven decision support tools to aid in the diagnosis and treatment of rare diseases.

**Fig. 6:**
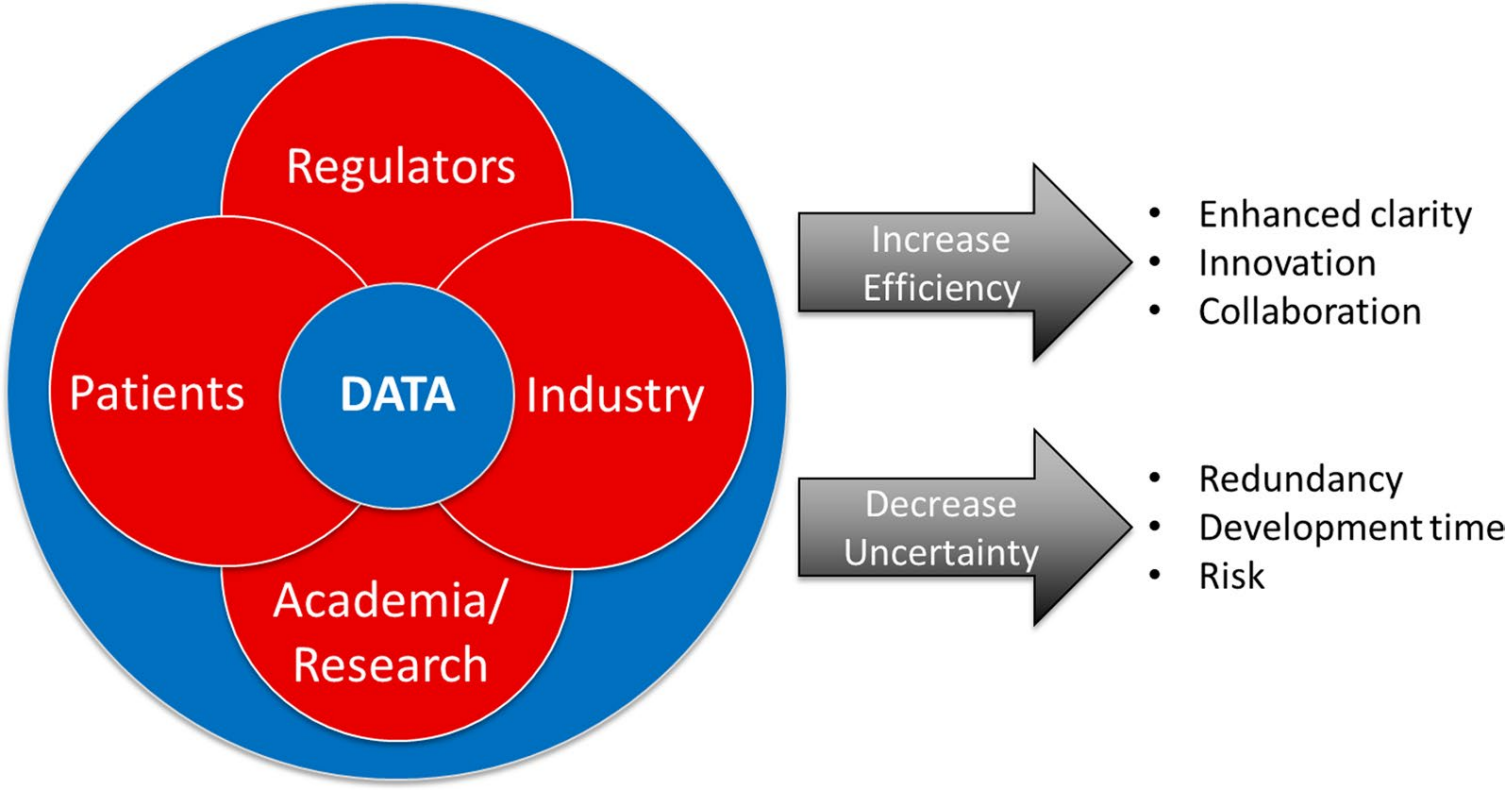
Multi-stakeholders involvement in providing data to advance diagnostics and therapies for rare disease patients. This graphic shows the requirement for different type of data originating from multiple stakeholder sources (patients, academic research, industry and regulators) and the need to share this information to efficiently advance the development of diagnoses and therapies

To truly ascertain the data that we propose is needed, it requires various stakeholders. As noted above and in Fig. 5, the need for different types of data is essential for advancing diagnostics and treatments in rare diseases. This data will come from a variety of sources including patients/families, research databases, electronic medical records, and health insurance information, most of which is held by different stakeholders (Fig. 6). Therefore, it is important that the various stakeholders are willing and able to share data and allow bi-directional flow of the data to maximize interpretation. It should be noted that the major sources of data are HCS and insurers. Both entities, whether through electronic medical records or reimbursement processes, have detailed information on patients: medical history, disease progression, treatments and interventions, as well as outcomes. Unfortunately, HCS and insurers can be reluctant to share data thus hindering advances in diagnostics and treatments. It is therefore important to demonstrate to these stakeholders the value of improved diagnostics and treatments on patient management, costs, and overall outcomes in order to encourage their participation in data sharing aimed at improving RD patient care.

The WG3 identified data elements that need to be considered for measuring how diagnosis and therapies impact the socio-economic burden on patients and families, and the economy and efficiency of HCS. These data elements can be categorized into 4 broad areas: Diagnostics, Prevalence, Natural History Studies, and Intervention. These are described in greater detail below and summarized in Table 3

- Diagnostic
  - Patient age, time to diagnose and quality of the diagnostic.
  - e.g. pediatric populations need an accurate diagnostic made at birth or in a short time (less than a year) after disease manifestation. Failure to diagnose early on will negatively impact the clinical intervention, increase the socio-economic burden on patients and families as well as the economic costs on the HCS.
- Prevalence
  - Measure of prevalence at birth, number of patients, age distribution and mortality.
  - e.g. measuring the prevalence of diseases is required to plan appropriately for their health care needs and is also useful clinically by providing context for diagnostic decision-making. Lack of data on disease prevalence will impair the time and quality of diagnosis, delay the development of proper health care resources, increase the economic costs on HCS and increase the socio-economic burden on patients and families.
- Natural history studies
  - Evaluation of the burden, disease progression models, disease registries, patients reported outcomes.
  - e.g. collection of real-world evidence data and conduct of qualitative natural history studies is essential for understanding the progression of a disease, developing its management strategy and supporting the development of safe and effective drugs and biological products for rare diseases. The lack of qualitative natural history data will negatively impact the clinical decision-making and therefore increase the economic costs on HCS and the socio-economic burden on patients and families.
- Intervention
  - Patient-centered outcome measure (PCOM), standard of care, alternative treatment, informed treatment response.
  - e.g. patient-centered outcome measures are essential for characterizing patient unmet needs and designing effective clinical trial studies and intervention leading to the development of data-driven support decision tools and new therapies for rare diseases. Failure to design effective clinical intervention will impede the clinical management of the diseases and therefore negatively impact the socio-economic burden on patients as well as the economic costs on HCS. Conversely, PCOMs are also important in understanding the potential negative impact of prohibitively expensive interventions on the social, emotional, and economic burden on RD patients and their families. This highlights another key issue in RD, namely that even when a diagnosis is made, the intervention can still be challenging and filled with just as many obstacles as the diagnostic journey itself.

**Table 3:** Essential disease-related data

Diagnostic	Age: Pediatric or adult Time to diagnose: Early (− 1 year) or late (+ 1 year) Quality of diagnosis: No diagnosis, misdiagnosis, diagnosis
Prevalence	Measure of prevalence Total number of patients Age distribution Mortality
Natural History Studies	Disease registries Evaluation of the burden Patient reported outcomes: Dynamic registries Disease progression models
Intervention	Patient-Centered Outcome Measures (IRDiRC Task Force) Standard of care Alternative/Innovative treatment Informed treatment response: Regulatory and effectiveness data

## Conclusion

In the world of rare diseases and the often long diagnostic odyssey that patients endure, it is evident that new tools are needed to improve the overall patient experience, identify new treatments, and reduce the socio-economic burden of rare diseases. As such, the work of IRDiRC in establishing working groups adds tremendous value to solving some of the greatest challenges facing rare disease patients. Through these working groups, IRDiRC provides an important venue through which groups of international stakeholders can convene and discuss essential topics to advance the diagnosis and treatment of rare diseases. The IRDiRC WG3 has developed recommendations on the metrics that are necessary for the development of new tools that will allow for faster and more accurate diagnosis, and to assess the overall impact of diagnosis and therapies on rare disease patients. These recommendations have been presented above and form the basis for continued collaborative work amongst the international rare disease community as we work towards a collective goal to “*Enable all people living with a rare disease to receive an accurate diagnosis, care, and available therapy within one year of coming to medical attention”* as defined by the IRDiRC Consortium.

## Data Availability

Not applicable.
